# Subpixel Localization of Isolated Edges and Streaks in Digital Images

**DOI:** 10.3390/jimaging6050033

**Published:** 2020-05-18

**Authors:** Devin T. Renshaw, John A. Christian

**Affiliations:** Department of Mechanical, Aerospace, and Nuclear Engineering, Rensselaer Polytechnic Institute, Troy, NY 12180, USA; renshd@rpi.edu

**Keywords:** image processing, edge localization, streak localization, Zernike moments, subpixel

## Abstract

Many modern sensing systems rely on the accurate extraction of measurement data from digital images. The localization of edges and streaks in digital images is an important example of this type of measurement, with these techniques appearing in many image processing pipelines. Several approaches attempt to solve this problem at both the pixel level and subpixel level. While the subpixel methods are often necessary for applications requiring best-possible accuracy, they are often susceptible to noise, use iterative methods, or require pre-processing. This work investigates a unified framework for subpixel edge and streak localization using Zernike moments with ramp-based and wedge-based signal models. The method described here is found to outperform the current state-of-the-art for digital images with common signal-to-noise ratios. Performance is demonstrated on both synthetic and real images.

## 1. Introduction

Digital images frequently contain valuable information about the real-world objects observed by a camera, telescope, or other optical system. This information may be used by a sensing system to understand, interpret, monitor, or analyze the properties of objects contained within the scene. Oftentimes, such systems attempt to distill the dense and complex information content of an image into a sparse set of simple and descriptive primitives—with edges and streaks being especially common examples. Making use of these primitives requires knowledge of their location in the image. Although pixel-level edge/streak localization is often adequate, some applications demand higher accuracy and motivate the need for subpixel localization.

Pixel-level edge localization is a ubiquitous image processing task, with a variety of techniques that can be found in almost every introductory text on image processing. Some popular classical methods are those of Sobel [1], Prewitt [2], Marr–Hildreth [3], and Canny [4], although there are many more. Motivated largely by problems in image segmentation, there has also been recent interest in edge detection and localization using deep learning [5], with notable contemporary examples including DeepEdge [6], DeepContour [7], holistic edge detector (HED) [8], and crisp edge detection (CED) [9].

There are also several methods available for subpixel edge localization. Many of these subpixel methods operate by refining a pixel-level edge guess into a subpixel-level estimate. The approaches for achieving such a subpixel correction vary, but generally belong to one of four different categories: moment-based [10,11], least-squares fitting [12], partial area effect [13], and interpolation [14].

In this work, we treat both edges and streaks as a local (and not global) concept, with each being identifiable by the 2D intensity pattern within a small image patch around a particular image point. Edge points are generally identified by finding pixels possessing a large intensity gradient (attempting to describe image points where there is thought to be an intensity discontinuity). A streak point is identified by finding pixels belonging to a bright (or dark) 1D path against a dark (or bright) background. In either case (edges or streaks), we seek only to localize isolated edge/streak points in this work.

This work presents a localization framework that is equally suitable to finding the subpixel location of edge and streak points within a digital image. Our method belongs to the moment-based category of techniques. We refine recent work on improved edge localization with Zernike moments [15] and then extend this approach to the closely related problem of streak localization. Unlike conventional subpixel edge localization methods using Zernike moments that assume an intensity step function [11], we model the underlying edge intensity function as a linear ramp. We use the same approach to model the underlying streak intensity function as a triangular wedge. This is the first application of Zernike moments (that the authors know of) to the subpixel localization of streaks in a digital image. The framework presented here is computationally efficient, non-iterative, and can be used within most imaging pipelines.

The remainder of this work is organized as follows. Section 2 introduces the coordinate frames and scaling conventions that are used in Section 3 to construct Zernike moments on local image patches. Section 4 describes how to use these Zernike moments for the subpixel localization of both edges and streaks. Performance of this approach is then demonstrated quantitatively on synthetic images (Section 5) and qualitatively on real images (Section 6).

## 2. Coordinate Frames and Conventions

Suppose that we have a digital image with *N* rows and *M* columns, with pixel intensity values stored in a N×M array (for a monochrome image). Define the *u* − *v* coordinate system with the origin in the upper lefthand corner such that pixel centers occur at integer values of *u* and *v*. The *u*-direction is to the right (corresponding to column number) and the *v*-direction is down (corresponding to the row number). We presume in this work that a different algorithm (e.g., Sobel [1], Canny [4]) has already produced pixel-level estimates for either an edge or streak location. Assuming such an algorithm has detected *m* such pixel locations, we denote the set of pixel-level guesses as ${\left\{{\tilde{u}}_{i},{\tilde{v}}_{i}\right\}}_{i}^{m}\subset {Z^{*}}^{2}$ (where $Z^{*}$ is the set of non-negative integers).

The algorithms presented in this work use a small image patch (e.g., 5×5 or 7×7) centered about a pixel-level estimate of an edge or streak location to compute a small correction to that feature’s location. The result is subpixel-level localization of a point belonging to an edge or streak. Furthermore, the moment-based methods to be discussed in Section 3 require the signal to be contained within the unit circle. Thus, data within each small image patch must be scaled to lie within the unit circle. For the *i*-th patch,

(1) $$\begin{array}{c} \bar{u}=\frac{2}{N_{p}}\left(u-{\tilde{u}}_{i}\right)\;\;\;\;\;\;\bar{v}=\frac{2}{N_{p}}\left(v-{\tilde{v}}_{i}\right), \end{array}$$

where $N_{p}$ is the size of the image patch (e.g., $N_{p}=5$ is a 5×5 patch). We generally constrain $N_{p}$ to be an odd integer, such that the pixel-level guess occurs at the center of the patch. This scaling ensures that $∥\bar{u}∥\leq 1$ and $∥\bar{v}∥\leq 1$ for every point within the square patch, and that ${\bar{u}}^{2}+{\bar{v}}^{2}\leq 1$ within the inscribed circle.

**Figure 1:**
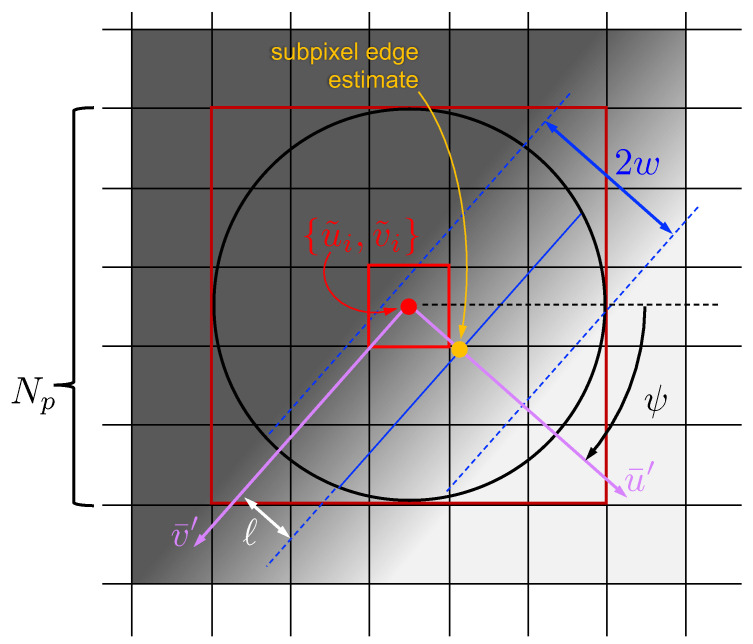
Example geometry of a square image patch ($N_{p}=5$, shown in dark red) centered about pixel-level edge guess $\left\{{\tilde{u}}_{i},{\tilde{v}}_{i}\right\}$ shown in bright red. The edge has a blur width of 2w and is offset from the pixel-level guess by a distance *ℓ*. The primed frame (rotated by an angle ψ relative to the unprimed image frame) is aligned with the edge, with ${\bar{v}}^{\prime }$ being parallel to the edge and ${\bar{u}}^{\prime }$ being normal to the edge. Although this figure shows only an edge, these coordinate frame conventions are the same for both edges and streaks.

We also find it convenient to define a rotated version of the $\bar{u}$ − $\bar{v}$ coordinate frame with an orientation dictated by the local normal of the edge or streak. Define a frame with coordinate axes ${\bar{u}}^{\prime }$ and ${\bar{v}}^{\prime }$ that are rotated by an angle ψ relative to the unprimed frame (Figure 1) such that the ${\bar{u}}^{\prime }$ direction is parallel with the local edge/streak normal and ${\bar{v}}^{\prime }$ is parallel to the edge/streak tangent. The direction ${\bar{u}}^{\prime }$ is chosen to be positive in the direction from dark to bright for an edge. Alternatively, for streaks, the positive ${\bar{u}}^{\prime }$ direction is chosen to be from the patch center towards the streak’s center. Thus, by construction, the correction from the pixel-level guess to the subpixel streak location is a small positive update along the ${\bar{u}}^{\prime }$ direction. The subpixel update along the ${\bar{u}}^{\prime }$ direction may be either positive or negative for an edge.

## 3. Computation of Zernike Moments in Digital Images

It is well-established that image moments are a useful tool for compactly describing the shape of the 2D intensity pattern within an image patch using only a small number of parameters. In general, 2D moments are a weighted average of the 2D signal value, with the weights for a particular moment coming from its corresponding basis function. That is, given a basis function $P_{nm}\left(u,v\right)$, the corresponding moment of the arbitrary 2D signal f(u,v) is computed as

(2) $$\;\int \int \;P_{nm}\left(\bar{u},\bar{v}\right)f\left(\bar{u},\bar{v}\right)\;d\bar{u}\;d\bar{v}$$

Since we will be computing moments within small image patches, we have chosen to express all functions in terms of the scaled pixel coordinates $\left\{\bar{u},\bar{v}\right\}$ as defined in Equation (1).

The choice of basis functions $P_{nm}$ is somewhat arbitrary, although it is desirable that the chosen set is both complete and orthogonal. In the case of edge or streak localization, we are looking for basis function sets defined within the unit disk. If $P_{nm}$ is chosen to be a polynomial in two variables, there are an infinite number of complete orthogonal sets [16], with the Zernike polynomials being the most commonly used.

### 3.1. Zernike Polynomials

Zernike polynomials, originally developed to aid in the study of spherical aberrations in optical lenses [17], have since found uses for a broad array of applications [11,15,18,19,20,21,22]. The Zernike polynomials may be written in either Cartesian or polar coordinates, with the polar form being the most commonly used [11],

(3) $$\begin{array}{c} P_{nm}\left(\rho ,\theta \right)=R_{nm}\left(\rho \right)\;\exp\left(jm\theta \right) \end{array}$$

where $j=\sqrt{-1}$ and

(4) $$\rho^{2}={\bar{u}}^{2}+{\bar{v}}^{2}$$

(5) $$R_{nm}\left(\rho \right)=\sum_{s=0}^{(n-|m|)/2}\frac{{\left(-1\right)}^{s}\left(n-s\right)!\;\rho^{n-2s}}{s!\;\left(\frac{n+|m|}{2}-s\right)!\;\left(\frac{n-|m|}{2}-s\right)!}$$

These polynomials form a complete set over a continuous space contained within the unit circle. The 1D radial polynomials, $R_{nm}\left(\rho \right)$, and their corresponding 2D Zernike polynomials, $P_{nm}\left(u,v\right)$, may be computed for a few common combinations of *n* and *m*,

(6)R00(ρ)=1⇒P00(u¯,v¯)=1(7)R11(ρ)=ρ⇒P11(u¯,v¯)=u¯+jv¯(8)R20(ρ)=2ρ2−1⇒P20(u¯,v¯)=2u¯2+2v¯2−1(9)R22(ρ)=ρ2⇒P22(u¯,v¯)=u¯2+v¯2

where the order *n* and repetition *m* [15] (or angular dependence [23]) can assume any values that satisfy

(10) $$\begin{array}{c} n\geq |m|\geq 0, \end{array}$$

(11) $$\begin{array}{c} n-m\;\;even. \end{array}$$

It is straightforward to show that Zernike polynomials are orthogonal under an $L_{2}$-inner product,

(12) $${\langle P_{\left(\alpha \right)},P_{\left(\beta \right)}\rangle }_{L_{2}}=\underset{u^{2}+v^{2}\leq 1}{\;\int \int \;}P_{\left(\alpha \right)}P_{\left(\beta \right)}^{*}\;du\;dv=Q_{\left(\alpha \right)}\delta_{\alpha \beta }$$

where $P_{\left(\alpha \right)}$ and $P_{\left(\beta \right)}$ are two arbitrary polynomials of the set, $P_{\left(\beta \right)}^{*}$ is the complex conjugate of $P_{\left(\beta \right)}$, and $\delta_{\alpha \beta }$ is the Kronecker delta function. Additionally, $Q_{\left(\alpha \right)}$ is the normalization coefficient and may be computed as [23]

(13) $$\begin{array}{c} Q_{nm}=\frac{\pi }{n+1} \end{array}$$

### 3.2. Zernike Moments for a Continuous 2D Signal

Zernike moments are formed by using the Zernike polynomials from Equation (3) as the basis functions in the 2D moment equation (Equation (2)). We express such a moment as

(14) $$\begin{array}{c} Z_{nm}=\frac{1}{Q_{nm}}\underset{u^{2}+v^{2}\leq 1}{\;\int \int \;}\;P_{nm}\left(u,v\right)\;f\left(u,v\right)\;du\;dv \end{array}$$

although we often find that scaling with the normalization coefficient is not required,

(15) $$\begin{array}{c} A_{nm}=\underset{u^{2}+v^{2}\leq 1}{\;\int \int \;}\;P_{nm}\left(u,v\right)\;f\left(u,v\right)\;du\;dv, \end{array}$$

This, of course, leads to the simple scaling relation

(16) $$\begin{array}{c} Z_{nm}=A_{nm}/Q_{nm} \end{array}$$

### 3.3. Rotational Properties of Zernike Moments

Zernike moments of repetition m=0 are rotationally invariant, as the value of the moment $A_{nm}$ is unaffected by the orientation of the underlying signal relative to the $\bar{u}$ − $\bar{v}$ coordinate system. For other values of *m* (i.e., for m>0), we find that the moment $A_{nm}$ changes as the orientation of the underlying signal changes.

Consider, for example, the moment $A_{nm}$ for a particular image patch as computed in the $\bar{u}$ − $\bar{v}$ frame. Now consider the moment $A_{nm}^{\prime }$ for this same image patch as computed in the ${\bar{u}}^{\prime }$ − ${\bar{v}}^{\prime }$ frame that has been rotated by an angle ψ relative to the unprimed frame (see Figure 1). Noting that $\theta^{\prime }=\theta -\psi$, it is clear from Equations (3) and (15) that

(17) $$A_{nm}^{\prime }=A_{nm}\exp\left(-jm\psi \right)$$

It is this relation that will ultimately allow us to determine the orientation of an edge or streak from the moment $A_{11}$.

### 3.4. Zernike Moments for a Digital Image

A digital image, I(u,v), is a quantized representation of the continuous signal f(u,v). The image I(u,v) is presumed to be an array of digital numbers, with integer intensity values (e.g., 0–65,535 for a 16-bit image) occurring at integer values of *u* and *v*.

In this case, we approximate the Zernike moment integral from Equation (15) with a double summation. Therefore, assuming a local image patch of size $N_{p}\times N_{p}$ centered at a pixel-level edge/streak guess of $\left\{{\tilde{u}}_{i},{\tilde{v}}_{i}\right\}$, one may compute the moment as

(18) $$A_{nm}\left({\tilde{u}}_{i},{\tilde{v}}_{i}\right)\approx \sum_{k=-p}^{p}\sum_{s=-p}^{p}I\left({\tilde{u}}_{i}+s,{\tilde{v}}_{i}+k\right)M_{nm}\left(p+s,p+k\right)$$

where $p=(N_{p}-1)/2$ is a non-negative integer (since $N_{p}$ is an odd integer greater than one). The mask $M_{nm}$ is an $N_{p}\times N_{p}$ matrix of values found by the integration of $P_{nm}$ over the corresponding pixel and within the patch’s inscribed circle. Values of $M_{11}$ and $M_{20}$ are shown for a 5×5 and 7×7 mask in [15]. It is observed that Equation (18) is simply an image correlation, such that one may compute the moment everywhere in the image according to

(19) $$A_{nm}=M_{nm}*I$$

where ∗ is the 2D correlation operator.

The edge and streak localization methods presented here will ultimately only use the moments $A_{11}$ and $A_{20}$. Of note is that $M_{20}$ is real valued such that

(20) $$A_{20}=M_{20}*I$$

We observe, however, that $M_{11}$ is complex valued,

(21) M11=ReM11+jImM11

Fortunately, given the structure of $M_{11}$, one only needs to keep track of the real component in practice since [15]

(22) ReA11=ReM11∗I

(23) ImA11=ReM11T∗I

Thus, we may compute all the necessary moments through three simple image correlations (which, in practice, only need to be computed at the pixel-level edge or streak locations and not at every point in the image).

## 4. Moment-Based Edge and Streak Localization

The same procedure may be used for both edge and streak localization. In both cases, the image data in a small $N_{p}\times N_{p}$ image patch around a pixel-level edge/streak guess is scaled according to Equation (1) and the Zernike moments $A_{11}$ and $A_{20}$ are computed (Equations (20), (22) and (23)). These moments are used to compute the edge/streak orientation (ψ) and the distance along this direction by which the pixel-level edge/streak guess should be adjusted (*ℓ*). Consequently, both the edge and the streak are corrected to subpixel accuracy by

(24) $$\begin{array}{c} \left[\begin{array}{c} \bar{u}\\ \bar{v} \end{array}\right]=\ell \left[\begin{array}{c} \cos\psi \\ \sin\psi \end{array}\right] \end{array}$$

which, after rearranging Equation (1), yields the correction we seek in practice

(25) $$\begin{array}{c} \left[\begin{array}{c} u\\ v \end{array}\right]=\left[\begin{array}{c} \tilde{u}\\ \tilde{v} \end{array}\right]+\frac{N_{p}\;\ell }{2}\left[\begin{array}{c} \cos\psi \\ \sin\psi \end{array}\right] \end{array}$$

The orientation ψ of both edges and streaks is found in the same way and using the same equation. The difference between the edge and streak correction is simply how the Zernike moments are used to compute *ℓ*.

### 4.1. Computing Edge or Streak Orientation

Determining the normal direction to an edge or streak is achieved in the exact same manner, with the final equation being equivalent for both. By construction, and as can be seen from Figure 1, the intensity value is only a function of ${\bar{u}}^{\prime }$ (i.e., not a function of ${\bar{v}}^{\prime }$) for both the edge and the streak. We see immediately from the form of $P_{11}$ in Equation (6) that

(26) ImA11′=0

Thus, recalling that exp(−jmψ)=cos(mψ)−jsin(mψ), we may rewrite Equation (17) as (for m=n=1)

(27) $$A_{11}^{\prime }=A_{11}\left[\cos(\psi )-j\sin(\psi )\right]$$

such that

(28) Re[A11′]=Re[A11]cos(ψ)+Im[A11]sin(ψ)

(29) Im[A11′]=Im[A11]cos(ψ)−Re[A11]sin(ψ)=0

The streak orientation may be found using the equation for the imaginary component of $A_{11}^{\prime }$. Observing that

(30) Im[A11]cos(ψ)=Re[A11]sin(ψ)

we find that the orientation of the streak is computed in terms of the moment $A_{11}$ (computed using Equations (22) and (23)) as

(31) ψ=atan2Im[A11],Re[A11]

This relation has been known for some time for edges [11,15]. Although obvious within the present framework, this represents the first extension of Equation (31) to streaks (of which the authors are aware).

### 4.2. Computing *ℓ* for Edges

An edge is generally understood to describe a discontinuity in image intensity in one direction, with little intensity change in the direction orthogonal to this discontinuity. Real grayscale images, however, rarely exhibit a true intensity discontinuities. Instead, image blur and pixel quantization cause the intensity change rapidly over a small distance (a few pixels). Thus, we seek areas of high intensity gradient rather than true discontinuities. It has long been known [10] that using a step function for the edge model within a moment-based subpixel edge localization algorithm produces a biased edge update if the image is blurred. This was one of the motivations for introducing a ramp edge model in [15].

**Figure 2:**
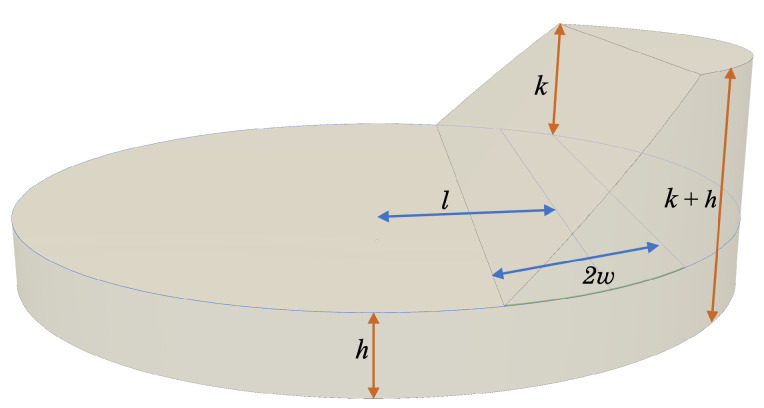
Graphical representation of a continuous edge signal (modeled as a linear ramp using Equation (34)) within the unit circle, including background intensity *h*, peak intensity of edge *k*, edge width *w*, and distance from the origin to the midpoint of the edge *ℓ*.

In many practical image processing problems, the point spread function (PSF) due to camera defocus and other optical effects is well modeled as a 2D Gaussian [24]. Consequently, the streak associated with a crisp edge (a true discontinuity) may be blurred according to

(32) $$I_{blur}\approx K_{G}*I$$

where *I* is the perfectly crisp image, $K_{G}$ is the Gaussian kernel, and $I_{blur}$ is the blurred image. The one-dimensional intensity profile taken perpendicular to the edge is sometimes referred to as the edge spread function (ESF), which will generally take the shape of a sigmoid function. To avoid the mathematical complexities of the sigmoid function within the Zernike moment integrals, it was observed in [15] that a linear ramp provides an adequate engineering approximation for most practical cases. The objective, therefore, is to relate the width of the linear ramp (2w full-width, see Figure 2) with the width of the Gaussian kernel approximating the camera PSF (σ). We do this using the linear relationship

(33) $$w\approx k_{edge}\sigma$$

where $k_{edge}$ is the scaling we seek. In [15], it was suggested to select $k_{edge}=1.66$. We performed a more comprehensive study and found that choosing $k_{edge}=1.80$ produced superior performance, especially as the SNR became very large. In general, we found reduced sensitivity to the choice of $k_{edge}$ as the images became noisier (lower SNR).

Therefore, we choose to model an edge as a ramp, whose intensity changes linearly between a background intensity (*h*) and a foreground intensity (h+k). The midpoint of this transition is defined to occur at a distance *ℓ* from the image patch center and has a width of 2w. Since we are using Zernike moments, we define all these quantities within the unit disk (Figure 2). By choosing to define the edge in the ${\bar{u}}^{\prime }$ − ${\bar{v}}^{\prime }$ frame, it is straightforward to write the intensity as a function of ${\bar{u}}^{\prime }$ only,

(34) $$I_{edge}\left({\bar{u}}^{\prime },{\bar{v}}^{\prime }\right)=\left\{\begin{array}{cc} h & {\bar{u}}^{\prime }\leq \ell -w\\ h+k\frac{{\bar{u}}^{\prime }-\left(\ell -w\right)}{2w} & \ell -w<{\bar{u}}^{\prime }\leq \ell +w\\ h+k & \ell +w<{\bar{u}}^{\prime } \end{array}\right.$$

Using this ramp edge model, it is possible to analytically solve the double integral in the moment equation from Equation (15) in the edge-aligned (i.e., primed) frame. We do this for the moments $A_{11}^{\prime }$ and $A_{20}^{\prime }$, leading to

(35) $$\begin{array}{c} \begin{array}{c} A_{11}^{\prime }=\frac{k}{24w}[3\arcsin\ell_{2}+\left(5-2\ell_{2}^{2}\right)\ell_{2}B_{2}\\ -3\arcsin\ell_{1}-\left(5-2\ell_{1}^{2}\right)\ell_{1}B_{1}] \end{array} \end{array}$$

(36) $$\begin{array}{c} A_{20}^{\prime }=A_{20}=\frac{k}{15w}\left[B_{1}^{5}-B_{2}^{5}\right] \end{array}$$

where

(37) $$\begin{array}{c} \ell_{1}=\ell -w\;\mathrm{and}\;\ell_{2}=\ell +w \end{array}$$

and

(38) $$\begin{array}{c} B_{1}=\sqrt{1-\ell_{1}^{2}}\;\mathrm{and}\;B_{2}=\sqrt{1-\ell_{2}^{2}} \end{array}$$

Looking at the expressions for $A_{11}^{\prime }$ and $A_{20}^{\prime }$, it is immediately evident that the the intensity-dependent variable *k* (which describes the magnitude of the intensity change across the edge) cancels out if one considers the ratio, $Q_{E}$

(39) $$\begin{array}{c} Q_{E}=\frac{A_{20}^{\prime }}{A_{11}^{\prime }}=\frac{A_{20}}{A_{11}^{\prime }} \end{array}$$

In many cases, the edge width *w* is known (e.g., from the imaging system point spread function), such that $Q_{E}$ is a function of only *ℓ*. Although the analytic expression for $Q_{E}$ is rather cumbersome, it was found in [15] that

(40) $$\begin{array}{c} Q_{E}\approx \ell \left[1-\left(1+\ell /2\right)w^{2}\right] \end{array}$$

which may be rearranged to solve for the unknown *ℓ*

(41) $$\begin{array}{c} {\hat{\ell }}_{E}\approx \frac{1-w^{2}-\sqrt{{\left(w^{2}-1\right)}^{2}-2w^{2}Q_{E}}}{w^{2}} \end{array}$$

Note that the ratio $Q_{E}$ is easy to compute in practice from the raw image moments found in a digital image,

(42) QE=A20′A11′=A20A11′=A20Re[A11]cosψ+Im[A11]sinψ

where $A_{20}$ from Equation (20), Re[A11] from Equation (22), Im[A11] from Equation (23), and ψ from Equation (31). Thus, with *w* and $Q_{E}$ known, Equation (41) may be used to solve for ${\hat{\ell }}_{E}$ for a given image patch.

### 4.3. Computing *ℓ* for Streaks

**Figure 3:**
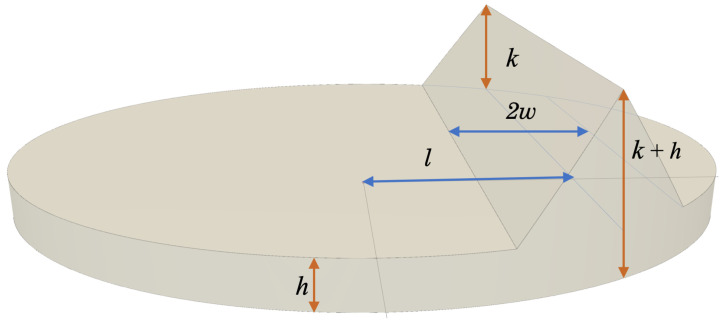
Graphical representation of a continuous streak signal (modeled as a wedge using Equation (44)) within the unit circle, including background intensity *h*, peak intensity of streak *k*, width of the streak *w*, and distance from the origin to the streak *ℓ*.

As a natural extension to the ideal step-function ESF, we model the ideal line spread function (LSF) as an impulse (where the LSF is defined as the 1D intensity profile perpendicular to the streak). As before, the perfectly crisp image is blurred with a Gaussian kernel, thus spreading out the line intensity, with the resulting LSF being Gaussian PDF. Rather than deal with the mathematical complexities of the Gaussian PDF, we choose to model the streak PSF as a wedge. To make practical use of the wedge model, it is necessary to determine the relationship between the wedge width (*w*, see Figure 3) and the Gaussian kernel width (σ),

(43) $$w\approx k_{streak}\sigma$$

where $k_{streak}$ is the parameter we seek. We found that choosing $k_{streak}=0.90$ provided the best results, with low SNR images exhibiting less sensitivity to the exact choice of this parameter.

The small image patch centered about the pixel-level guess is assumed to have a constant background intensity of *h* and contain a streak of intensity h+k. The wedge has a full width of 2w with a peak intensity occurring at a distance *ℓ* from the image patch (or disk) center. The sides of the wedge are linear ramps transitioning between the background and the streak’s ridgeline. This is shown pictorially on the unit disk in Figure 3.

As with the edge, we choose to define the streak model in the ${\bar{u}}^{\prime }$ − ${\bar{v}}^{\prime }$ frame such that the intensity is a function of ${\bar{u}}^{\prime }$ only (and not a function of ${\bar{v}}^{\prime }$),

(44) $$I_{streak}\left({\bar{u}}^{\prime },{\bar{v}}^{\prime }\right)=\left\{\begin{array}{cc} h & {\bar{u}}^{\prime }\leq \ell -w\\ h+k\frac{{\bar{u}}^{\prime }-\left(\ell -w\right)}{w} & \ell -w<{\bar{u}}^{\prime }\leq \ell \\ h+k-k\frac{{\bar{u}}^{\prime }-\ell }{w} & \ell <{\bar{u}}^{\prime }\leq \ell +w\\ h & \ell +w<{\bar{u}}^{\prime } \end{array}\right.$$

The analytical value of $A_{11}^{\prime }$ and $A_{20}^{\prime }$ may be found by evaluating the double integral from Equation (15)

(45) $$\begin{array}{c} \begin{array}{c} A_{11}^{\prime }=\frac{k}{12w}[6\arcsin\ell -3\arcsin\ell_{1}-3\arcsin\ell_{2}\\ +2C\ell (5-2\ell^{2})\;\;\\ -B_{1}\ell_{1}\left(5-2\ell_{1}^{2}\right)\;\;\\ -B_{2}\ell_{2}\left(5-2\ell_{2}^{2}\right)] \end{array} \end{array}$$

and

(46) $$\begin{array}{c} \begin{array}{c} A_{20}^{{}^{\prime }}=\frac{2k}{15w}\left[B_{1}^{5}+B_{2}^{5}-2C^{5}\right], \end{array} \end{array}$$

where $B_{1}$ and $B_{2}$ are from Equation (38) and

(47) $$\begin{array}{c} C=\sqrt{1-\ell^{2}} \end{array}$$

The ratio of $A_{20}^{\prime }$ to $A_{11}^{{}^{\prime }}$ eliminates *k*, thus providing a function of only *ℓ* and *w*,

(48) $$\begin{array}{c} Q_{S}\left(\ell ,w\right)=\frac{A_{20}^{\prime }}{A_{11}^{\prime }}=\frac{A_{20}}{A_{11}^{\prime }} \end{array}$$

Assuming the streak width *w* is known, we seek to rearrange $Q_{S}$ to solve for the unknown *ℓ*. The complicated form of $Q_{S}$ after substitution of Equation (45) and Equation (46) makes finding an analytic solution difficult for arbitrary values of *w* and *ℓ*. Fortunately, it is straightforward to find an approximation that is good enough for most practical image processing applications.

We know that streaks are thin, so it is instructive to explore what happens to $Q_{S}$ as w→0. We find that the limit does permit a simple analytic solution,

(49) $$\begin{array}{c} Q_{S_{0}}=\lim_{w\to 0}Q_{S}=\frac{4\ell_{S_{0}}^{2}-1}{3\ell_{S_{0}}} \end{array}$$

which may be solved for for $\ell_{S_{0}}$

(50) $$\begin{array}{c} {\hat{\ell }}_{S_{0}}=\frac{3}{8}Q_{S_{0}}\pm \sqrt{{\left(\frac{3}{8}Q_{S_{0}}\right)}^{2}+\frac{1}{4}} \end{array}$$

To choose the correct root, observe that

(51) $$\begin{array}{c} \lim_{\ell_{S_{0}}\to 0^{+}}Q_{S_{0}}=\lim_{\ell_{S_{0}}\to 0^{+}}\frac{4\ell_{S_{0}}^{2}-1}{3\ell_{S_{0}}}=-\infty \end{array}$$

where we know to choose the right limit since $\ell_{S}\geq 0$ by construction. Thus we seek the root that is approximately zero when $Q_{S_{0}}$ is a large negative number, which only happens when the plus sign is chosen in Equation (50). Therefore,

(52) $$\begin{array}{c} {\hat{\ell }}_{S_{0}}=\frac{3}{8}Q_{S_{0}}+\sqrt{{\left(\frac{3}{8}Q_{S_{0}}\right)}^{2}+\frac{1}{4}} \end{array}$$

This analytic result may be generalized to the situation where w>0, which does not appear to permit an exact analytic solution. Therefore, we write a parameterized expression for ${\hat{\ell }}_{S}$ that simplifies exactly to the form of Equation (52) when w=0 and fit the parameters in a least squares sense. Using this approach, consider a model of the form

(53) $$\begin{array}{c} \begin{array}{c} {\hat{\ell }}_{S}\approx \sqrt{a_{1}^{2}Q_{S}^{2}+a_{2}Q_{S}w+a_{3}w^{2}+a_{4}w+a_{5}}+a_{6}Q_{S}+a_{7}w+a_{8} \end{array} \end{array}$$

We found the terms associated with $a_{1}$, $a_{3}$, $a_{5}$, and $a_{6}$ to dominate the estimate of $\ell_{S}$, with the remaining terms contributing relatively little. Furthermore, it was found that $a_{1}\approx a_{6}$ regardless of the test set-up. Therefore, discarding the unimportant terms and letting $a_{1}=a_{6}$, we performed a three parameter fit for the streak correction of the form

(54) $$\begin{array}{c} \begin{array}{c} {\hat{\ell }}_{S}\approx \sqrt{a_{1}^{2}Q_{S}^{2}+a_{3}w^{2}+a_{5}}+a_{1}Q_{S}. \end{array} \end{array}$$

The result of the least squares fit found the values of $a_{1}$ and $a_{5}$ to exactly match the analytically derived coefficients for $\ell_{S_{0}}$ in Equation (52)

(55) $$\begin{array}{c} a_{1}=3/8,\;\;\;a_{5}=1/4 \end{array}$$

and empirically found that

(56) $$\begin{array}{c} a_{3}\approx -1/10 \end{array}$$

Therefore, we may write the empirically derived expression for the streak update for arbitrary *w* as

(57) $$\begin{array}{c} {\hat{\ell }}_{S}=\frac{3}{8}Q_{S}+\sqrt{{\left(\frac{3}{8}Q_{S}\right)}^{2}-\frac{1}{10}w^{2}+\frac{1}{4}} \end{array}$$

As with the edge update, note that the streak ratio $Q_{S}$ is easy to compute in practice from the raw image moments found in a digital image,

(58) QS=A20′A11′=A20A11′=A20Re[A11]cosψ+Im[A11]sinψ

where $A_{20}$ from Equation (20), Re[A11] from Equation (22), Im[A11] from Equation (23), and ψ from Equation (31). Thus, with *w* and $Q_{S}$ known, Equation (57) may be used to solve for ${\hat{\ell }}_{S}$ for a given image patch.

Observe that $Q_{E}$ and $Q_{S}$ are the same moment ratio, $A_{20}^{\prime }/A_{11}^{\prime }$; hence, the equations to compute these ratios from the raw image moments are the same (compare Equation (42) and Equation (58)). What differs is the assumption of the underlying signal (a ramp or an edge), leading to a different relationship (Equation (41) or Equation (57)) between the moment ratio and the subpixel location of the edge or streak.

## 5. Numerical Validation on Synthetic Images

The performance of the edge and streak localization methods presented in this work were quantitatively evaluated using synthetic images. We find synthetic images to be especially useful in this context since the true continuous location of every image feature is known. The perfectly known continuous underlying signal may be blurred to simulate camera defocus and quantized (both spatially and in intensity) to simulate differing image resolutions. Further, noise may be added with a prescribed intensity, allowing the unambiguous evaluation of performance as a function of signal-to-noise ratio (SNR). This is important, as the localization of edges and streaks is known to become more challenging as SNR decreases [25,26]. Of particular note is that our new streak localization method works for 1D paths of arbitrary shape, whereas most existing streak detection algorithms—especially for faint (low SNR) streaks—presume the streaks are straight lines.

For the examples presented here, perfect images were blurred by using a Gaussian point spread function (PSF). After blurring, zero-mean Gaussian noise was added to achieve the specified SNR.

### 5.1. Synthetic Images with Edges

#### 5.1.1. Ideal Edge Localization Performance

**Figure 4:**
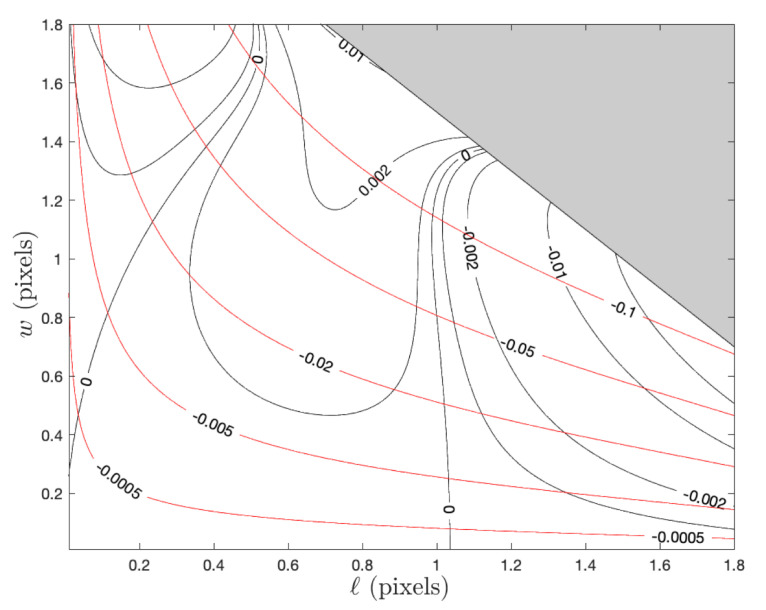
Contours of edge localization error for a continuous (not pixelated) ramp edge signal. Black contours show the error when using the approximation from Equation (41), red contours show the error when using the step function approximation from [11].

It is important to quantify the error associated with the approximations used to arrive at the analytic edge update given in Equation (41). Therefore, as a bounding case, suppose that we perfectly compute the Zernike moments for a noise-free continuous signal. In this situation, the error in ${\hat{\ell }}_{E}$ is given by the contours in Figure 4 for different situations. These contours visually demonstrate the performance improvement afforded by switching from the step-function edge model (red contours) to the ramp edge model (black contours). The results shown here are identical to the observations of Christian in [15].

#### 5.1.2. Digital Image Edge Localization Performance

**Figure 5:**
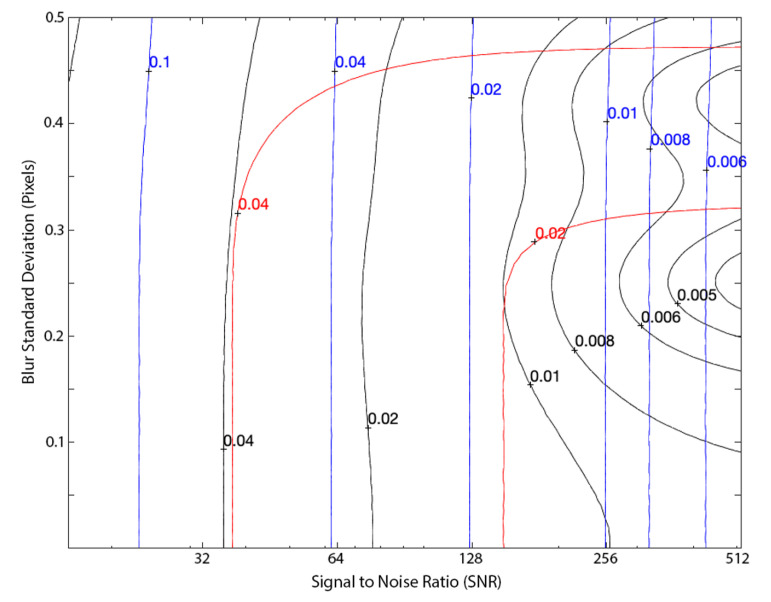
Contours of edge localization error (in pixels, assuming a 5×5 mask) in a digital image for our method from Equation (41) (black), the step function approximation using Zernike moments (red) [11], and the partial area effect (blue) [13] as a function of SNR and blur. Error statistics are computed from a Monte Carlo analysis consisting of 5000 randomized images at each SNR and blur combination.

Our method performed favorably to other existing techniques when processing synthetic digital imagery. This was assessed through a Monte Carlo analysis where we evaluated performance of different algorithms for images having varying amounts of blur and noise. Figure 5 shows edge localization error with our technique (black contours) compared against the moment-based solution with a step-function edge model [11] and the partial area effect (PAE) [13]. Results for both of the two moment-based methods shown here assume a 5×5 pixel mask.

Note that the PAE method from [13] was chosen as one of the two comparison methods in Figure 5 since this represents the current state-of-the-art. Indeed, this method has recently been used for the subpixel localization of edges in a wide variety of applications [27,28,29].

We observe that the PAE algorithm produced nearly perfect edge localization in cases with no noise (infinite SNR; off the right-hand side of Figure 5). The Zernike moment methods tended to perform better than the PAE method as noise increased (as SNR decreased; towards the left-hand side of Figure 5). The method presented in this work outperforms the PAE method for most real-life SNR values.

**Figure 6:**
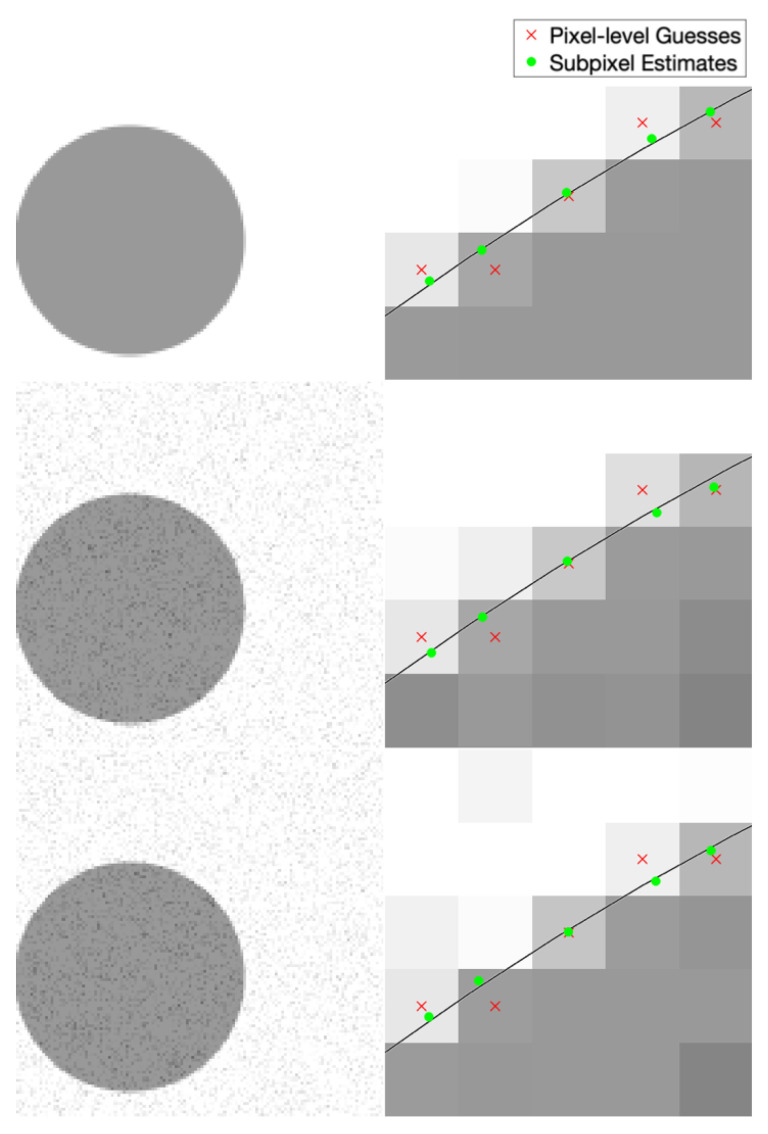
Qualitative visualization of subpixel edge localization performance at varying levels of blur and SNR. The left column shows the full synthetically generated image and the right column shows a small area within that image. The rows represent different noise and blur levels (top: no noise or blur; middle: noise only (approximately 28.4 peak signal to noise ratio); bottom: noise and blur (2D Gaussian kernel with standard deviation 0.3 pixels)). The black line is the exact location of the true edge.

Example performance of our subpixel edge localization algorithm in different noise/blur cases is shown in Figure 6. This example shows localization of the edge of a circle. Clear improvement is evident in all cases, as the algorithm moves the pixel-level edge guess (red ×) towards the true edge location (black line). We know the true edge location since these are synthetic images.

### 5.2. Synthetic Images with Streaks

#### 5.2.1. Ideal Streak Localization Performance

**Figure 7:**
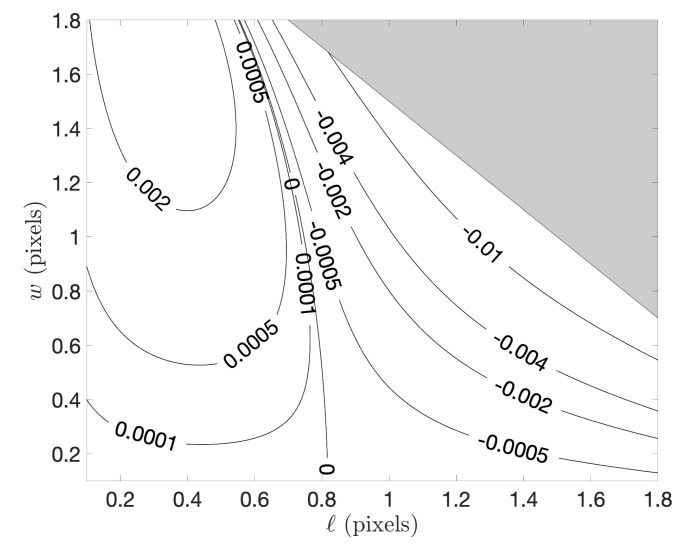
Contours of streak localization error when using Equation (57) for a continuous (not pixelated) wedge edge signal.

As with the case of edges, we begin the numerical assessment of our subpixel streak localization method by considering the case of a continuous signal. This allows us to directly quantify the error associated with the approximations used to arrive at the analytic expression in Equation (57). We considered all reasonably plausible combinations of streak location (*ℓ*) and streak width (*w*) and produced contours of errors in the estimate ${\hat{\ell }}_{S}$, as shown in Figure 7. These errors are low enough to be negligible when applied to a pixelated image.

#### 5.2.2. Digital Image Streak Localization Performance

**Figure 8:**
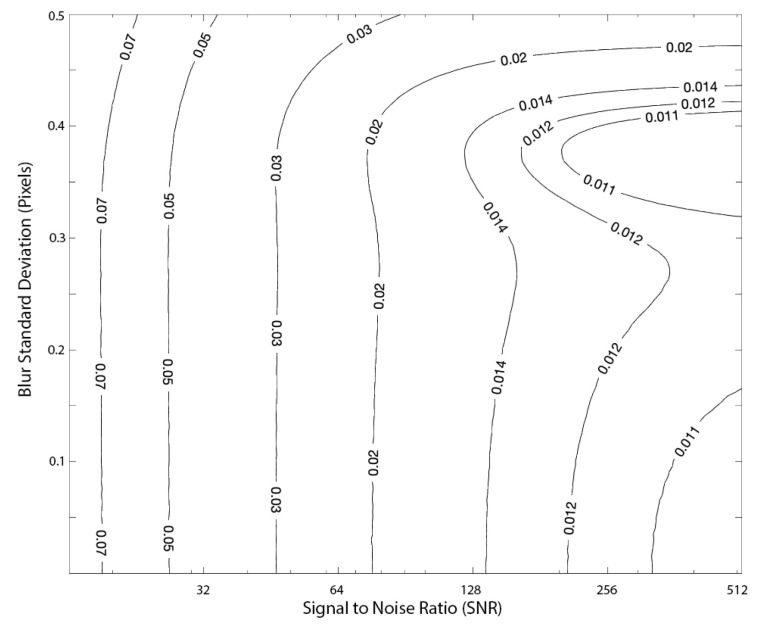
Contours of streak localization error (in pixels, assuming a 5×5 mask) in a digital image for our method as a function of SNR and blur. Error statistics are computed from a Monte Carlo analysis consisting of 5000 randomized images at each SNR and blur combination.

Our Zernike moment method also performed well in the subpixel localization of streaks. We performed a Monte Carlo analysis where streak localization error was recorded for varying amounts of image blur and noise. The results are shown as contours in Figure 8. As expected, localization performance decreases with increased noise and blur.

**Figure 9:**
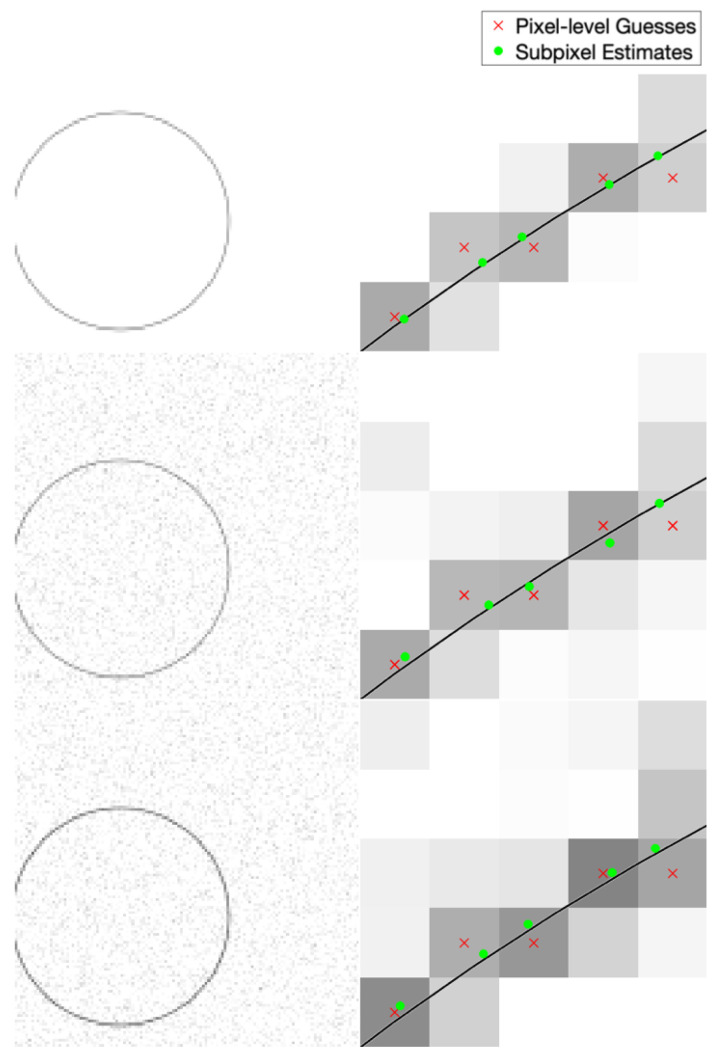
Qualitative visualization of subpixel streak localization performance at varying levels of blur and SNR. The left column shows the full synthetically generated image and the right column shows a small area within that image. The rows represent different noise and blur levels (top: no noise or blur; middle: noise only(approximately 28.5 peak signal to noise ratio); bottom: noise and blur (2D Gaussian kernel with standard deviation of 0.3 pixels)). The black line is the exact location of the true streak center.

Example performance of our subpixel streak localization algorithm in different noise/blur cases is shown in Figure 9. This example shows localization of a circular streak. Clear improvement is evident in all cases, as the algorithm moves the pixel-level streak guess (red ×) towards the true streak location (black line). We know the true streak location since these are synthetic images.

## 6. Validation on Real Data

After confirming that estimated edge and streak locations agree with the truth in simulated images, we apply our method to real digital images. As these real-world images do not provide perfect subpixel knowledge of the edge or streak location, verifying results is from visual inspection and is largely qualitative.

It is important to remember that the algorithm presented here only performs the subpixel localization (i.e., correction) on pixel-level location guesses (e.g., using Sobel [1], Canny [4], or other method); any edges or streaks that the higher-level algorithm fails to identify will not contribute to the final result. Note that these pixel-level guesses may be found automatically or manually. Regardless of how they are found, the subpixel correction discussed in this manuscript is automatic.

This section includes a number of example images with the accompanying results from the methods proposed in this paper. These examples show the raw image on the left-most frame, followed by two sections of the image in grayscale containing streaks or edges of interest (center and right frame). We highlight performance by progressively zooming in on a specific portion of the image (moving left to right), with blue boxes indicating the region-of-interest for the subsequent frame. The middle frame of each example only shows the subpixel estimate overlay (green dots). The right frame of each example shows both the pixel-level guess overlay (red ×) and the subpixel estimate overlay (green dots). The right frame also shows the edge or streak estimates connected by a line to help illustrate the improvement in smoothness naturally produced by the subpixel correction.

**Figure 10:**
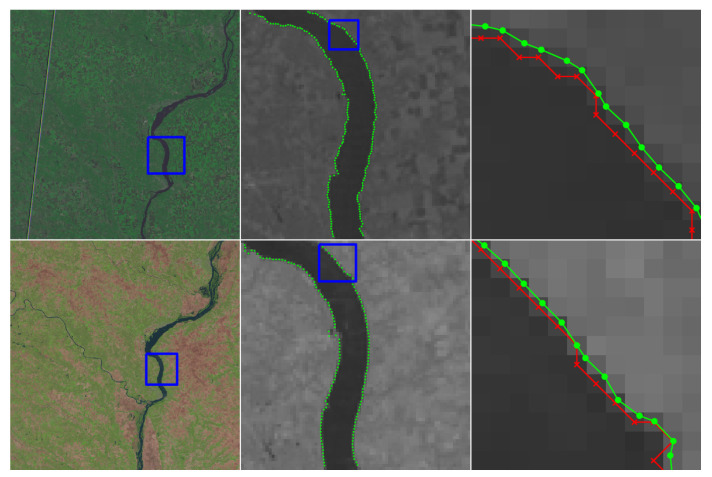
Images of the Mississippi River taken by the Landsat-5 spacecraft, where we seek to localize the river banks. The top image (LM05 L1TP_025032_20120830_20180521_01_T2) was collected on 21 May 2018 by the Multispectral Scanner System (MSS) and shows the river during normal conditions. The bottom image (LT05_L1TP_025032 20110508_20160902_01_T1) was collected on 2 September 2011 by the thematic mapper (TM) and shows the river after a major flooding event. The red × symbols denote pixel-level edge estimates and green dots denote the refined subpixel localization estimates. Image data is available from the U.S. Geological Survey (USGS) [30].

**Figure 11:**
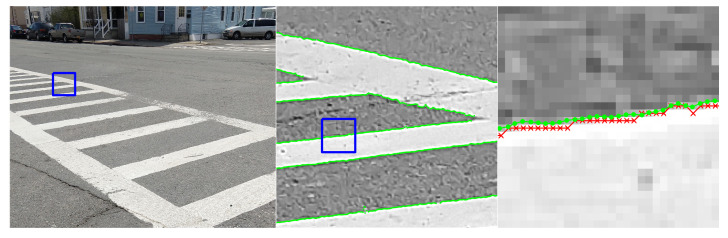
Image of a pedestrian crosswalk in Watervliet, NY, where we seek to localize the edges of the painted surface markings. The red × symbols denote pixel-level edge estimates and green dots denote the refined subpixel localization estimates. Original image collected by the authors with a personal camera.

**Figure 12:**
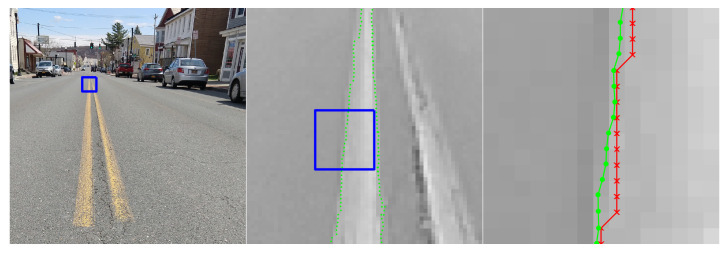
Image of a street in Watervliet, NY, where we seek to localize the edges of the painted yellow lane markings. The red × symbols denote pixel-level edge estimates and green dots denote the refined subpixel localization estimates. Original image collected by the authors with a personal camera.

**Figure 13:**
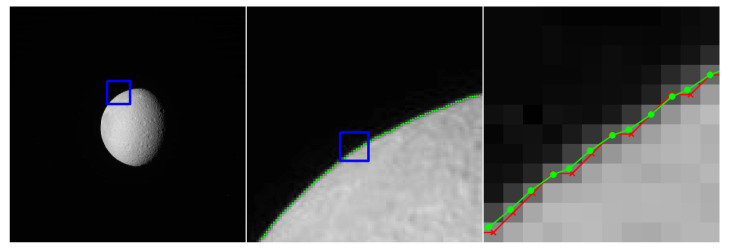
Image of Rhea (a moon of Saturn) collected by the Cassini spacecraft’s Narrow Angle Camera (NAC) on 13 October 2006 (raw image N1539252663 [31]), where we seek to localize the moon’s lit limb. The red × symbols denote pixel-level edge estimates and green dots denote the refined subpixel localization estimates.

**Figure 14:**
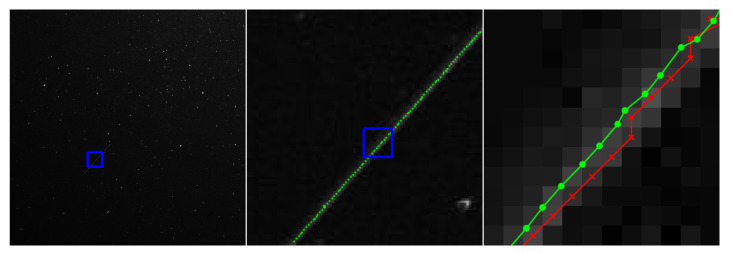
Inertially pointed star field image captured with with the Omnidirectional Space Situational Awareness (OmniSSA) system. This example image has a 10 s exposure time and contains a satellite that appears as a streak within the image. The red × symbols denote pixel-level streak estimates and green dots denote the refined subpixel localization estimates. The original OmniSSA image is courtesy of Dr. Marcus Holzinger of University of Colorado Boulder.

**Figure 15:**
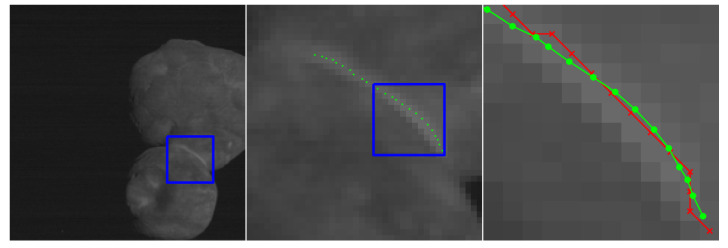
Image of Kuiper belt object Arrokoth (formerly called Ultima Thule) collected by the New Horizon spacecraft’s Long Range Reconnaissance Imager (LORRI) during a flyby in early 2019 (credit for raw image: NASA/Johns Hopkins University Applied Physics Laboratory/Southwest Research Institute). The red × symbols denote pixel-level streak estimates and green dots denote the refined subpixel localization estimates.

**Figure 16:**
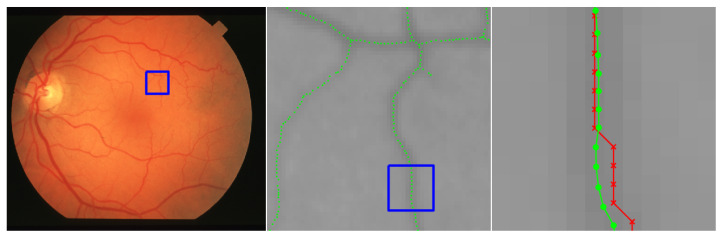
Image of a retinal scan for a healthy eye, where we seek to localize blood vessels. The red × symbols denote pixel-level streak estimates and green dots denote the refined subpixel localization estimates. The original image is im00032 from the STARE database [32,33].

**Figure 17:**
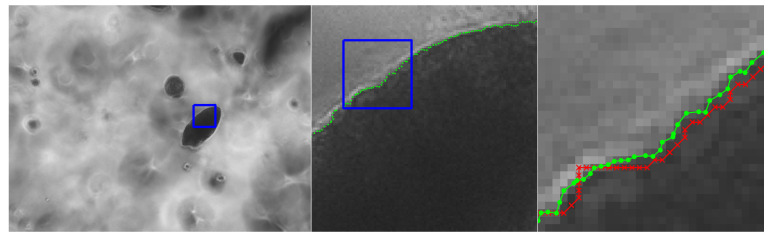
Microscope image from an in vitro tumor model embedded in a hydrogel. We seek to localize the edges of tumors to measure their growth over time [34,35]. The red × symbols denote pixel-level edge estimates and green dots denote the refined subpixel localization estimates. The original image is courtesy of Dr. Kristen Mills of Rensselaer Polytechnic Institute.

Figure 10 shows an application to natural disaster management that illustrates the difference in the shores of the Mississippi River in the aftermath of a flood (bottom) and its normal banks (top). Figure 11 and Figure 12 show the application of the proposed technique for the subpixel localization of common road surface markings (e.g., pedestrian crosswalk markings, lane markings). Figure 13, Figure 14 and Figure 15 show various applications to space exploration. Finally, Figure 16 and Figure 17 highlight the potential use of this method in medical imaging (e.g., tracing the routes of blood vessels in a retinal scan, microscope imaging of tumors). The diversity of example images is intended to emphasize that the techniques presented in this manuscript are application agnostic and can be applied to a wide variety of image processing tasks.

## 7. Conclusions

Many modern sensing systems rely on the accurate extraction of measurement data from digital images. The localization of edges and streaks in digital images is an important example of this type of measurement, with these techniques appearing in many image processing pipelines.

Zernike moments are powerful tools in image processing and have been used for subpixel edge localization for over 25 years. In this manuscript, we describe a new way to exploit Zernike moment data to produce subpixel edge estimates, resulting in improved localization performance relative to earlier techniques using Zernike moments to achieve this same task. We also show how this same framework can be extended to the task of subpixel localization of streak. As far as the authors know, this represents the first application of Zernike moments to subpixel streak localization.

Correcting a pixel-level guess of either an edge or a streak requires use of only two Zernike moments ($A_{11}$ and $A_{20}$), with both of these moments being computed over a small image patch centered about the pixel-level guess. One of the principal innovations of this work is the use of a linear ramp (for an edge) or triangular wedge (for a streak) signal model. These simplified models make it possible to refine the pixel-level guess to subpixel accuracy using an analytic function of these two moments and knowledge of the edge/streak width. Furthermore, we show this new method to be tolerant to noise and to outperform many existing methods. Performance was quantitatively evaluated on synthetic images (localization error less than 0.1 pixel for both edges and streaks) and qualitatively evaluated on real images. Applications were shown for remote sensing, localization of road markings, space exploration, and medical imaging.
